# Fatty Acid Composition, Oxidative Status, and Content of Biogenic Elements in Raw Oats Modified Through Agricultural Practices

**DOI:** 10.3390/foods13223622

**Published:** 2024-11-13

**Authors:** Michał Wojtacki, Krystyna Żuk-Gołaszewska, Robert Duliński, Joanna Giza-Gołaszewska, Barbara Kalisz, Janusz Gołaszewski

**Affiliations:** 1 Department of Agrotechnology and Agribusiness, Faculty of Agriculture and Forestry, University of Warmia and Mazury in Olsztyn, ul. Oczapowskiego 8, 10-718 Olsztyn, Poland; wojtacki.michal@gmail.com (M.W.); kzg@uwm.edu.pl (K.Ż.-G.); 2 Department of Biotechnology and General Food Technology, Faculty of Food Technology, University of Agriculture in Krakow, ul. Balicka 122, 30-149 Kraków, Poland; robert.dulinski@urk.edu.pl; 3 Department of Midwifery, Centre of Postgraduate Medical Education, ul. Żelazna 90, 01-004 Warszawa, Poland; joannagiza@interia.eu; 4 Department of Soil Science and Microbiology, Faculty of Agriculture and Forestry, University of Warmia and Mazury in Olsztyn, Pl. Łódzki 3, 10-719 Olsztyn, Poland; barbara.kalisz@uwm.edu.pl; 5 Department of Genetics, Plant Breeding and Bioresource Engineering, Faculty of Agriculture and Forestry, University of Warmia and Mazury in Olsztyn, Pl. Łódzki 3, 10-724 Olsztyn, Poland

**Keywords:** *Avena sativa*, agronomic practices, fatty acids, tocopherols, biogenic elements, dietary value

## Abstract

The chemical composition of raw oat grain is responsible for the high dietary value and health-promoting properties of oat products. This article presents the results of a study investigating the biofortification of grain in two oat genotypes—hulless and hulled—through agronomic treatments: chemical plant protection against weeds and fungi and mineral nitrogen fertilization. The applied agronomic treatments induced different changes in the fatty acid profiles, content of tocopherols, macronutrients, and micronutrients in the grain of hulled and hulless oats. Plant health contributed to higher concentrations of unsaturated fatty acids and potassium in oat grain. In turn, nitrogen fertilization decreased the content of unsaturated fatty acids, potassium, and copper and increased the content of saturated fatty acids, calcium, and manganese in oat grain. At the same time, agronomic treatments reduced the tocopherol content of the grain, which implies that the nutritional value of oats increases in the absence of chemical plant protection agents. The correlations between the content of desirable chemical compounds and agronomic treatments were stronger in hulless oat grain, which may suggest that the agronomic modification of oat-based foods is more effective in this genotype. The content of exogenous alpha-linoleic acid C18:3 n-3 and alpha-tocopherol was higher in grain harvested from the control treatment (without chemical plant protection), whereas grain harvested from fully protected treatments accumulated more essential gamma-linolenic acid C18:3 n-6. The content of gamma-tocopherol and copper in oat grain was higher in the absence of nitrogen fertilization.

## 1. Introduction

The common oat (*Avena sativa* L.) is unique among cereals due to its multifunctional ingredients that provide health benefits and nutritional properties [1]. Oat grain is characterized by high nutritional and energy values. Oat-based foods, including products recommended for vegetarians and vegans, have a high dietary value because they are abundant in slowly digestible starch and protein and have a high content of exogenous amino acids (including lysine), unsaturated fatty acids (UFAs), fiber (including β-glucan), minerals, and vitamins [2,3,4]. The protein content of oat grain ranges from 11% to 15% (on a dry matter basis). The protein profile of oats differs from that of other cereals, mainly due to a high content of globulins (80%) and a low content of prolamins (avenin) (15%), as well as the distribution of protein fractions in grain, including a high content of globulins (storage proteins) that are accumulated mainly in the endosperm [5]. Starch and lipids are a source of energy and UFAs. Oat grain is more abundant in starch and lipids than other cereals, and these components are accumulated mainly in the endosperm. The fat content of oat grain ranges from 4.9 to 10.5 g 100 g^−1^, and it accounts for 5–9% of total lipids. Unsaturated fatty acids account for 78–81.5% of total fatty acids in oat grains [6]. Keying et al. [7] analyzed changes in the lipid content of hulless oat (*Avena nuda* L.) grain and concluded that the prevention of lipid hydrolysis is an important consideration in the production of oat-based foods. They found that the lipid content of raw oat grain remained relatively stable after one year of storage, providing protection to endogenous antioxidants, including tocopherols, L-ascorbic acid, thiols, amino acids, and phenolic compounds.

The dietary value of oat products is determined by the content of dietary fibers and biogenic elements. Fiber is composed of many chemical compounds, including oligosaccharides, polysaccharides, lignin, and related compounds that are not digested in the small intestine but can be completely or partially fermented in the large intestine [8]. Oat grain is particularly abundant in β-glucan (2.3–8.5 g 100 g^−1^), a polysaccharide that is soluble in water and has health-promoting properties [9]. The content of minerals and their proportions determine the nutritional value of oat products [10]. Deng et al. [11] identified the main locations of macronutrients and micronutrients in grain. They found that Ca and Mn were stored mainly in the aleurone layer and the scutellum, whereas P, K, Fe, Cu, and Zn were accumulated mainly in the aleurone layer and the embryo. The concentrations of P, K, Cu, and Zn were higher in the scutellum than in other parts of the grain. The concentrations of S and Cu were highest in the nucellar projection of the crease region. The cited study provides important information for micronutrient biofortification and processing strategies in oats.

The health-promoting properties of oats are harnessed by the pharmaceutical industry to treat and alleviate various diseases, including endometriosis [12] and gluten intolerance in celiac disease. Gluten-free foods play a very important role in the treatment of these diseases. Oat grain contains avenin, a protein that is structurally similar to wheat, barley, and rye glutens. Avenin accounts for a small proportion of total oat proteins, which is why oat-based foods are generally regarded as gluten-free [13]. Research has shown that oat products are generally safe for consumers with celiac disease, although excessive consumption of oat avenin may cause adverse gastrointestinal symptoms in consumers who are allergic to gluten. According to Hardy et al. [14], oat intake in the human diet is generally insufficient to induce clinical flare-ups of celiac disease, and long-term feeding studies have demonstrated that oats are safe for this group of consumers. Oat products are highly recommended for infants and older people, not only because of their high nutritional value but also due to the lack of allergenicity, palatable flavor, long shelf-life, and stability [15]. Oat products exert a number of physiological effects that may be beneficial in the prevention or amelioration of pathophysiological states, including improvements in gastrointestinal function, modulation of glucose metabolism, and reduction of blood cholesterol levels [16,17]. Fardet [18] and Gil et al. [19] found that whole-grain oats reduced the risk of diabetes and cancer. Oat products exert hypocholesterolemic and hypotensive effects by regulating blood sugar levels and eliminating free radicals [20].

Oat grain contains natural antioxidants, including tocopherols, alk(en)ylresorcinols, phenolic acids, and their derivatives, and it is a unique source of avenanthramides with antihistaminic activity [21]. In addition, oats are a unique source of avenanthramides (N-cinnamoylanthranilate alkaloids) and avenalumic acids (ethylenic homologs of cinnamic acids) that are not found in other cereal grains [22,23,24,25]. In turn, dietary fiber prevents heart disease, diabetes, and some types of cancer, and it has been found to improve short-term and long-term memory. The World Health Organization (WHO) [26] recommends an intake of at least 25 g of dietary fiber per day. Diets deficient in dietary fibers contribute to obesity.

The quality of oat grain is determined by agricultural practices, including nitrogen fertilization [27,28], weed and fungi control, and treatments that promote plant health [29,30], as well as oat variety (genotype) [31,32]. Oat cultivars belong to two genotypes: hulled and hulless. Hulless oat cultivars are lower in fiber and contain significantly more energy, total lipids, linoleic acid, protein, and starch than hulled oat cultivars [31,33]. Hulless oat grain is more digestible due to a lower content of fiber [33]. Oat grain is characterized by high processing suitability and low processing costs. Hulled oats are easily dehulled, whereas hulless cultivars do not require this treatment [34]. Therefore, hulless oats are a rational choice in the production of grain with health-promoting properties, and appropriate agronomic treatments can modify the nutritional value of oat products [33].

The biofortification of dietary products has attracted significant research interest in recent years [35]. The present study was undertaken to examine the relationship between the fatty acid profiles, antioxidant status, and content of biogenic elements in raw hulled and hulless oat grain harvested from nine treatments with different combinations of crop protection strategies and nitrogen fertilization. Therefore, the objective of the study was to determine: (i) the influence of agronomic treatments on the composition of fatty acids, tocopherols, and biogenic elements in unprocessed grain of hulled and hulless oats; and (ii) the relationship between the analyzed grain components and agronomic treatments.

## 2. Materials and Methods

### 2.1. Plant Material

Oat grain samples for qualitative analyses were obtained from a field experiment with a three-factor design that was conducted in 2016. The environmental conditions were typical for a continental climate with a cold winter, no dry season, and a warm summer [36]. The experimental factors were chemical plant protection (control—no protection {C}, herbicide {H}, herbicide and fungicide {HF}), mineral nitrogen fertilization (at the rates of 0, 60, and 120 kg ha^−1^ {N0} {N60}, {N120}), and two oat morphotypes (hulless oat cv. Nagus and hulled oat cv. Nawigator).

### 2.2. Laboratory Analyses

#### 2.2.1. Proximate Analysis

Grain samples were cleaned and milled using a Wiley mill. The obtained flour was passed through a 0.5 mm mesh sieve and stored at 4 °C until analysis. The content of dry matter, crude ash, and crude fiber was determined in a proximate analysis according to AOAC standard methods [37].

#### 2.2.2. Fatty Acids

Fatty acids were identified using chromatographic separation [38]. Fatty acid methyl esters for the determination of fatty acid composition were prepared according to Peisker’s method with some modifications (methanol:chloroform:concentrated sulfuric acid, 100:100:1 *v*/*v*). Fatty acids were separated and identified with gas chromatography using the Agilent 6890N Network Gas Chromatograph (Agilent Technologies, Inc. Headquarters, Santa Clara, CA, USA) with a flame ionization detector (FID), a capillary column (length—30 m; internal diameter—0.32 mm), a liquid stationary phase, and 0.25 µm film thickness. The injection volume was 1 µL. The detector temperature was 250 °C, the injector temperature was 230 °C, and the column temperature was 195 °C. The carrier gas was helium with a flow rate of 1.5 mL/min, and the split ratio was 50:1. Fatty acids were identified by comparing their retention times with those of individual methyl ester standards (Sigma-Aldrich (Supelco), St. Luis, MO, USA) and peak retention times in the sample. The relative content of individual fatty acids was expressed as a percentage of the sum of all fatty acids in the sample.

The fatty acids identified in oat morphotypes are shown in Table 1. Alpha-linoleic acid (C18:3 n-3) and trace amounts (<0.1%) of osbond acid (C22:5 n-6) and clupanodonic acid (C22:5 n-3) were identified only in treatments without plant protection.

#### 2.2.3. Tocopherols

The content of alpha- (α-T), beta- (β-T), gamma- (γ-T), and delta-tocopherol (δ-T) in oat grain was determined at low light intensity. Grain samples were ground in a Retsch SK 100 laboratory mill (aperture size: 1 mm). Ground samples were weighed in two replicates (5 g); 30 cm^3^ of 20% (*w*/*v*) ascorbic acid (aqueous solution) was added (p.a., Sigma-Aldrich), and the mixture was extracted with 50 cm^3^ of petroleum ether:acetone (*v*/*v*, 1:1) (p.a., POCh) (24 h, in the dark, at room temperature). Next, the mixture was saponified through the addition of 5 cm^3^ of 50% (*w*/*v*) aqueous solution of KOH (p.a., POCh) (24 h, in the dark, at room temperature). Next, the samples were extracted with 1 × 50 cm^3^ of ethyl ether, followed by 3 × 30 cm^3^ of petroleum ether (40/60, p.a., POCh). Ether extracts were combined and rinsed with 10% (*w*/*v*) aqueous solution of NaCl (p.a., POCh), followed by deionized water. The eluates were dehydrated with anhydrous sodium sulfate (p.a., POCh) and evaporated to dryness on a rotary evaporator (40 °C) (Janke & Kunkel IKA–Labortechnik, IKA-WERKE, GMBH & CO, KG, Staufen, Germany). The dried residue was dissolved in 5 cm^3^ of n-hexane (p.a., POCh), passed through a PTFE syringe filter (0.22 μm), and analyzed in a Shimadzu HPLC system (RP-HPLC) equipped with a Nukleosil C_18_ column (250 × 4.6 mm, 5 μm) and an RF detector (E_x_ = 293, E_m_ = 326). Methanol:H_2_O (95:5 *v*/*v*) (HPLC grade, Sigma-Aldrich, St. Luis, MO, USA) was the mobile phase with a flow rate of 1 cm^3^ min^−1^, and the loop size was 20 µL. The results were interpreted based on external standards: (±)-alpha-tocopherol (DL-all-rac a-tocopherol), beta-tocopherol, (+)-gamma-tocopherol, and (+)-delta-tocopherol (Sigma-Aldrich (Supelco), St. Luis, MO, USA) [39].

#### 2.2.4. Biogenic Elements

To determine the content of nitrogen, analytical grain samples of 0.5 g each were wet mineralized in concentrated sulfuric acid (10 cm^3^) with the addition of hydrogen peroxide as the oxidizing agent. Each sample was transferred to a 100 cm^3^ volumetric flask and topped up with distilled water. In the prepared mixtures, the content of nitrogen was determined with sodium hypochlorite using a Shimadzu spectrophotometer (Shimadzu HPLC system (RP-HPLC) – Shimadzu UV 2600, Shimadzu Europa GmbH, Germany) [40]. The P content of the grain was determined by the vanadium-molybdate method using a Shimadzu spectrophotometer. To determine the content of K, Ca, Mg, and micronutrients (Cu, Fe, Zn, Mn), grain samples were digested in a mixture of nitric and perchloric acids with the addition of hydrochloric acid [41]. The concentrations of Mg, Cu, Fe, Zn, and Mn were determined by atomic absorption spectrometry (Shimadzu AA 6800), and the concentrations of K and Ca were determined by atomic emission spectrometry (FLAPHO flame photometer, Carl Zeiss Jena, Germany).

### 2.3. Statistical Analyses

The chemical properties of oat grain were processed statistically by the analysis of variance (ANOVA) to assess the significance of the main effects and interaction effects of agronomic treatments. Treatment means were compared using Tukey’s test. The relationships between agronomic treatments and the chemical properties of hulless and hulled oat grain were determined by the principal component analysis (PCA). All statistical analyses were performed at a significance level of *p* < 0.05.

## 3. Results and Discussion

### 3.1. Proximate Composition and Fatty Acid Profile

The main effects of agronomic treatments on the content of ash, fiber, fat, and fatty acids are presented in Table 2. The content of ash and fiber in grain was significantly differentiated by nitrogen fertilization and oat morphotype, whereas fat content was influenced only by oat morphotype. On average, increasing nitrogen rates decreased ash content by 11% and fiber content by 22%. In comparison with hulless oat, hulled oat grain contained 19% more ash and 3.5 times more fiber but 54% less fat. The predominant SFAs in oat grain were oleic acid C18:1 n-9 (41.8%) and linoleic acid C18:2 n-6 (30.2%), and the predominant UFA was palmitic acid C16:0 (22.2%); essential fatty acids (EFAs) accounted for 30.9% of total fatty acids in oat grain.

Nitrogen fertilization did not induce significant differences in the fatty acid content of grain, except for an increase in stearic acid (C18:0) concentration. In turn, plant protection treatments promoted the accumulation of UFAs, including alpha-linolenic acid (C18:3 n-3) and erucic acid (C22:1 n-9). Plant protection treatments induced a significant increase in the content of oleic acid (C18:1 n-9) and linoleic acid (C18:2 n-6) and a decrease in the content of palmitoleic acid (C16:1 n-7) and behenic acid (C22:0). These observations indicate that plant health promoted the accumulation of UFAs, including EFAs. It should be noted that the content of alpha-linoleic acid increased three-fold in response to herbicide application and 4.5-fold in response to the combined herbicide and fungicide treatment. Alpha-linoleic acid is an EFA that is not synthesized by the human body and acts as a substrate for the production of other UFAs with health-promoting properties, including eicosapentaenoic, docosapentaenoic, and docosahexaenoic acids [42]. In comparison with hulled oat, hulless oat grain contained significantly more oleic acid (C18:1 n-9) (44.6% vs. 39.1%), stearic acid (C18:0) (2.255% vs. 1.523%) and arachidic acid (C20:0) (0.187% vs. 0.153%), but less myristic acid (C14:0) (0.260% vs. 0.369%), palmitoleic acid (C16:1 n-7) (0.255% vs. 0.297%), and vaccenic acid (C18:1 n-7) (0.997% vs. 1.165%).

**Figure 1:**
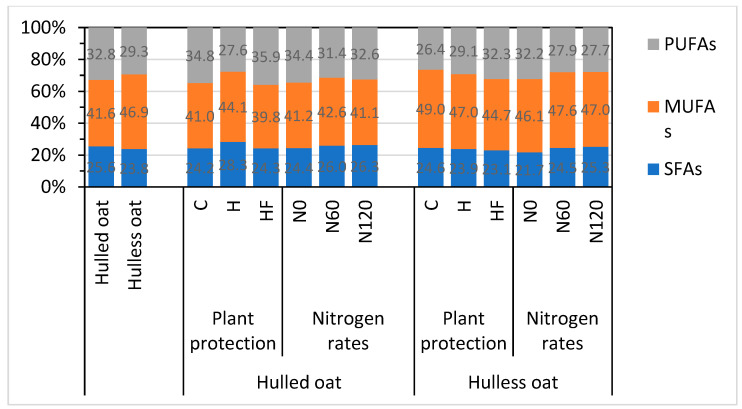
Groups of fatty acids in the grain of different oat morphotypes, and *oat morphotype* x *plant protection* and *oat morphotype* x *nitrogen fertilization* interactions. C—control without plant protection; H—herbicide; HF—herbicide and fungicide; N0, N60, N120—nitrogen rate of 0, 60, and 120 kg ha^−1^, respectively; SFAs—saturated fatty acids; MUFAs—monounsaturated fatty acids; PUFAs—polyunsaturated fatty acids.

In general, the analyzed oat morphotypes did not differ significantly in the content of saturated fatty acids (SFAs) and polyunsaturated fatty acids (PUFAs), whereas the content of monounsaturated fatty acids (MUFAs) was around 13% higher in hulless than hulled oat grain (Figure 1). The fatty acid analysis validated previous observations that healthy oat plants synthesize more UFAs. In hulless oat grain, the content of PUFAs was 10% higher in the herbicide treatment and 22% higher in the herbicide + fungicide treatment, relative to the control treatment.

### 3.2. Tocopherols

**Figure 2:**
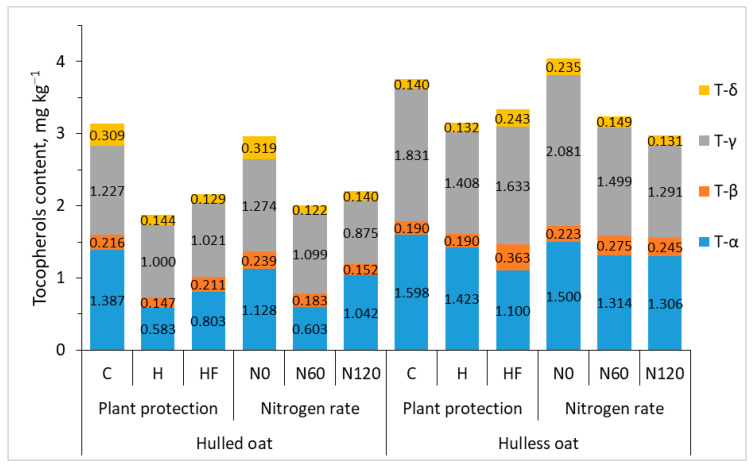
Interaction effects of agronomic treatments on the content of tocopherol in the grain of hulled and hulless oats. C—control without plant protection; H—herbicide; HF—herbicide and fungicide; N0, N60, N120—nitrogen rates of 0, 60, and 120 kg ha^−1^, respectively.

In plants, tocopherols participate in many physiological processes and increase plant resistance to abiotic stresses [43]. These compounds also play an important role in the human diet and are used in the prevention and treatment of various diseases. Tocopherols are lipophilic antioxidants that scavenge reactive oxygen species and prevent lipid peroxidation [44]. Tocopherol isoforms α, β, γ, and δ differ in their antioxidant capacity. In the current study, all experimental factors influenced the total tocopherol content of oat grain, which varied considerably from 1.509 to 4.631 mg 100 g^−1^ depending on the agronomic treatment. Plant protection and nitrogen fertilization decreased tocopherol content by around 25% on average, and total tocopherol content was 42% higher in hulless than hulled oat grain (Table 3, Figure 2). In general, agronomic treatments exerted similar effects on total tocopherol content and the content of each isoform in oat grain.

Alpha-tocopherol (α-T), one of the eight isoforms of vitamin E (four tocopherols and four tocotrienols), is the most powerful, fat-soluble natural antioxidant. Vitamin E scavenges lipophilic peroxyl radicals, in particular oxidized low-density lipoproteins, and confers protection against atherosclerosis. Vitamin E also delivers pro-oxidant effects and participates in cell signaling and gene regulatory functions [45]. In the present study, the content of α-T varied considerably from 0.424 to 2.049 mg 100 g^−1^ (1.143 mg 100 g^−1^ on average). The α-T content of grain was 33% lower in protected treatments. This parameter was significantly higher (by 49%) in hulless (1.374 mg kg^−1^) than in hulled oat grain (0.924 mg 100 g^−1^).

Gamma-tocopherol (γ-T) is the main isoform of vitamin E that delivers health-promoting effects and decreases the risk of cardiovascular diseases and cancer [46]. Gamma- and α-T were the predominant tocopherols in oat grain, and the content of γ-T was 52% higher in hulless than in hulled oat grain.

Beta-tocopherol (β-T) is a less potent antioxidant than T-α, and it accounted for only 7–8% of total tocopherols in oat grain. The concentration of delta-tocopherol (δ-T) was also relatively low (5–8% of total tocopherols). Delta-tocopherol’s mechanism of action in oats has not been fully elucidated to date, but research has shown that δ-T scavenges free radicals and protects cells against oxidative damage. Li et al. [47] reported that δ-T effectively reduced oxidative damage to DNA, inhibited nitrotyrosine formation, and enhanced apoptosis in cancer cells. In turn, γ-T exhibited weaker anti-cancer effects, whereas α-T demonstrated no such activity. In the current study, the average content of δ-T was higher in the grain of hulled than hulless oat, but the difference was not statistically significant.

### 3.3. Biogenic Elements

The analysis of the main effects of agronomic treatments (Table 4) revealed that chemical plant protection increased the content of potassium (by 6%) and decreased the content of phosphorus (by 17%), calcium (by 23%), and zinc (16%) in oat grain. In turn, nitrogen fertilization significantly increased the content of calcium (by 80%) and manganese (by 13%) but decreased the concentrations of potassium (by 9%) and copper (by 28%).

**Figure 3:**
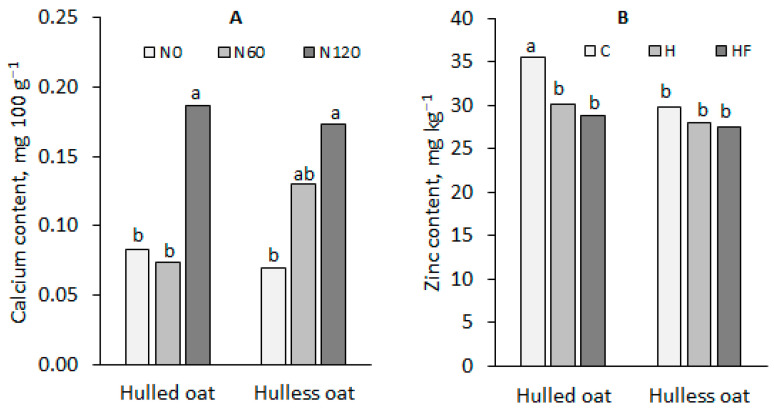
Significant effects of (**A**) the *nitrogen fertilization* x *oat morphotype* interaction on the calcium content of oat grain and (**B**) the *plant protection* x *oat morphotype* interaction on the zinc content of oat grain. The same lower-case letters next to the bars indicate a statistically insignificant difference according to Tukey’s test.

In comparison with hulled oat, the grain of hulless oat was characterized by a significantly higher content of phosphorus (18%) and magnesium (14%) and a lower content of iron (26%), zinc (10%), and manganese (10%). It should also be noted that the calcium content was significantly influenced by the *nitrogen fertilization* x *oat morphotype* interaction, and the content of zinc was significantly affected by the plant protection x oat morphotype interaction (Figure 3).

The calcium content of hulled oat grain increased significantly only in response to the nitrogen rate of 120 kg ha^−1^, whereas in hulless oat grain, this effect was observed already after the application of 60 kg N ha^−1^. Plant protection treatments tended to decrease zinc content in the grain of both oat morphotypes, but a significant reduction was noted only in hulled oat grain.

### 3.4. The Relationship Between Agronomic Treatments and the Chemical Properties of Hulless and Hulled Oat Grain

**Figure 4:**
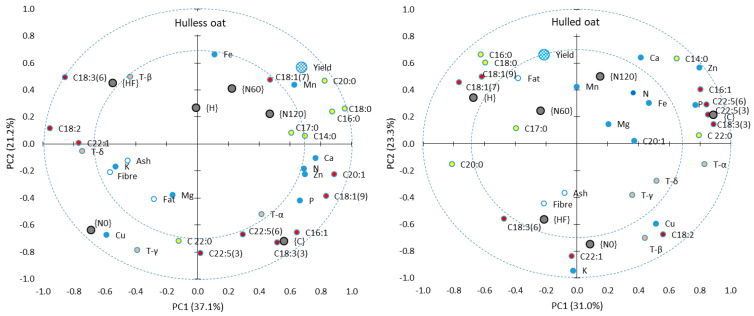
Principal component analysis (PCA)—the first two principal components (PC1 and PC2) describe the relationships between agronomic treatments and the chemical properties of hulless (**left**) and hulled oat (**right**) grain. Inner and outer dotted circles represent correlation coefficients of 0.7 and 1, respectively. Black circles denote agronomic treatments: {C}—control without chemical plant protection, {H}—herbicide application, {HF}—herbicide and fungicide application, {N0}—without mineral nitrogen fertilization, {N60} and {N120}—nitrogen fertilization applied at 60 and 120 kg ha^−1^, respectively. Blue circles—macronutrients and micronutrients. Yellow circles—saturated fatty acids. Red circles—unsaturated fatty acids. White circles—proximates. Gray circles—tocopherols.

In the PCA, the first two principal components explained more than 50% of the total variance in both oat morphotypes, which indicates that the applied agronomic treatments exerted similar effects on the chemical properties of oat grain (Figure 4). However, in hulless oats, desirable changes in the chemical properties of grain were more highly correlated with specific agronomic treatments. In both oat morphotypes, grain yield (Y) was positively correlated with nitrogen fertilization ({N60} and {N120}), the concentrations of UFAs and vaccenic acid (C18:1 n-7) (MUFA). Interestingly, the absence of nitrogen fertilization {N0} was strongly correlated with gamma-tocopherol (γ-T) and copper levels in hulless oat grain and with the content of paullinic acid (C22:1) and potassium content in hulled oat grain.

In hulless oat grain, the absence of plant protection {C} was strongly correlated with the content of SFAs: palmitoleic acid (C16:1), alpha-linoleic acid (C18:3 n-3), osbond acid (C22:5 n-3), clupanodonic acid (C22:5 n-6), and alpha-tocopherol (α-T). In hulled oat grain, the absence of plant protection was strongly correlated with all of the above parameters as well as the content of UFAs: myristic acid (C14:0) and behenic acid (C22:0) (UFAs).

In both oat morphotypes, herbicide and fungicide application {HF} was correlated with the content of gamma-linoleic acid (C18:3 n-6). Full plant protection was also correlated with the content of beta-tocopherol (β-T) in hulless oat grain and with the content of ash and fiber in hulled oat grain. Herbicide application {H} was not correlated with any chemical parameter in hulless oat grain, but it was correlated with the content of palmitic acid (C16:0), stearic acid (C18:0) (UFAs), vaccenic acid (C18:1 n-7), oleic acid (C18:1 n-9) (MUFAs), and fat in hulled oat grain.

## 4. Summary and Conclusions

The analysis of the effects of agronomic treatments on the chemical composition of oat grain revealed that specific treatments induced different changes in the dietary quality of oat products. Chemical plant protection and mineral nitrogen fertilization significantly modified the chemical composition of oat grain. In addition, hulled and hulless oat grain was characterized by specific combinations of chemical compounds in response to the applied agronomic treatments.

Hulless oat grain contained more fat (54%) but less ash (19%) and fiber (3.5-fold) than hulled oat grain. Oleic acid (C18:1 n-9), linoleic acid (C18:2 n-6), and palmitic acid (C16:0) accounted for 94% of total fatty acids, whereas EFAs (C18:2 n-6, C18:3 n-3, C18:3 n-6) accounted for 31% of total fatty acids in oat grain. The compared oat morphotypes had a similar content of SFAs and PUFAs, but the content of MUFAs was 13% higher in hulless oat grain. On average, the content of PUFAs in oat grain increased by 10% in response to herbicide application and by 22% in response to combined herbicide and fungicide application.

On average, total tocopherol content was 43% higher in hulless than in hulled oat grain. The concentrations of γ-T and α-T were 49% higher, and the content of β-T was 30% higher in hulless oat grain. The applied agronomic treatments decreased tocopherol levels in oat grain.

The analysis of the macronutrient and micronutrient content of oat grain revealed that chemical plant protection increased potassium levels but decreased the content of phosphorus, calcium, and zinc in oat grain. In turn, nitrogen fertilization increased calcium and manganese concentrations but decreased potassium and copper levels in grain. Hulless oat grain was characterized by a significantly higher content of phosphorus (18%) and magnesium (14%) and a lower content of iron (26%), zinc (10%), and manganese (10%). The calcium content of grain was influenced by the interaction effects of oat morphotype and increasing nitrogen rates, whereas zinc levels were affected by the interaction effects of oat morphotype and plant protection treatments.

In conclusion, the study demonstrated that hulless oat has a higher potential for agronomic modification due to stronger correlations between the concentrations of valuable grain nutrients and specific agronomic treatments. In hulless oat grain, the content of exogenous alpha-linoleic acid (C18:3 n-3) and alpha-tocopherol peaked in the absence of chemical plant protection. In turn, full chemical plant protection increased the concentration of exogenous gamma-linolenic acid (C18:3 n-6) in the grain of both oat morphotypes. The absence of nitrogen fertilization was correlated with the content of gamma-tocopherol and copper in hulless oat grain, and with the content of erucic acid (C20:1 n-7) and potassium in hulled oat grain. These observations indicate that the absence of chemical plant protection and mineral fertilization can enhance the content of valuable nutrients in oat grain. However, oat yields are significantly reduced in organic farming systems.

## Figures and Tables

**Table 1:** Composition of fatty acids identified in oat grain.

Symbol (*nx*: the Double Bond Is Located on the *x*th C–C Bond)		Systematic Name	Common Name
Saturated fatty acids (SFAs)			
C14:0		tetradecanoic acid	myristic acid
C16:0		hexadecanoic acid	palmitic acid
C17:0		heptadecanoic acid	margaric acid
C18:0		octadecanoic acid	stearic acid
C20:0		eicosanoic acid	arachidic acid
C22:0		docosanoic acid	behenic acid
Monounsaturated fatty acids (MUFAs)			
C16:1 n-7		hexadecenoic acid	palmitoleic acid
C18:1 n-7		octadecenoic acid	vaccenic acid
C18:1 n-9		octadecenoic acid	oleic acid
C20:1 n-7		eicosenoic acid	paullinic acid
C22:1 n-9		erucic acid	erucic acid
Polyunsaturated fatty acids (PUFAs)			
C18:2 n-6	Essential fatty acid (EFA)	octadecadienoic acid	linoleic acid
C18:3 n-3	Essential fatty acid (EFA)	octadecatrienoic acid	alpha-linolenic acid
C18:3 n-6	Essential fatty acid (EFA)	octadecatrienoic acid	gamma-linolenic acid
C22:5 n-6		docosapentaenoic acid	osbond acid
C22:5 n-3		docosapentaenoic acid	clupanodonic acid

**Table 2:** Main effects of agronomic treatments on the chemical properties of oat grain—proximate analysis and composition of fatty acids. Mean values marked with different letters are statistically significant according to Tukey’s test.

Chemical Properties	Unit	Plant Protection			Nitrogen Rate			Oat Morphotype	
		Control	Herbicide	Herbicide and Fungicide	N_0_	N_60_	N_120_	Hulled Oat	Hulless Oat
Proximate analysis									
Dry matter	%	91.1	90.9	91.1	91.3	90.7	91.1	91.1	90.9
Ash	%DM	2.028	2.133	2.057	2.195 a	1.943 b	2.08 ab	2.254 a	1.891 b
Fiber	%DM	5.710	6.310	5.673	6.800	5.612	5.282	9.212 a	2.583 b
Fat	%DM	5.215	5.052	5.065	5.348	4.922	5.062	4.020 b	6.201 a
Fatty acids †									
C14:0 †	%	0.340	0.303	0.301	0.290	0.301	0.352	0.369 a	0.260 b
C16:0	%	21.8	23.5	21.3	20.8	22.7	23.2	23.3 a	21.1 b
C16:1	%	0.384 a	0.230 b	0.214 b	0.266	0.284	0.278	0.297 a	0.255 b
C17:0	%	0.054	0.056	0.055	0.050	0.053	0.062	0.054	0.056
C18:0	%	1.860	1.904	1.705	1.651	1.915	1.902	1.523 b	2.123 a
C18:1 (n-9)	%	42.4	43.2	39.8	41.2	42.8	41.5	39.1 b	44.6 a
C18:1 (n-7)	%	1.046	1.093	1.074	1.031	1.094	1.088	1.165 a	0.977 b
C18:2 (n-6)	%	29.4	27.8	33.2	32.3	28.8	29.3	31.8	28.5
C18:3 (n-6)	%	0.193 c	0.587 b	0.887 a	0.635	0.510	0.523	0.573	0.539
C18:3 (n-3) ‡	%	0.240	ND ‡	ND	0.272	0.210	0.239	0.306	0.175
C20:0	%	0.163	0.176	0.171	0.159	0.180	0.171	0.153 b	0.187 a
C20:1 (n-7)	%	1.057	0.895	0.940	0.972	0.887	1.034	0.960	0.969
C 22:0	%	0.205	0.095	0.120	0.152	0.142	0.126	0.177	0.103
C22:1 (n-9)	%	0.096 b	0.102 b	0.207 a	0.181	0.102	0.122	0.150	0.120
C 22:5 (n-6) ‡	%	0.054	ND	ND	0.044	0.057	0.062	0.087	0.022
C 22:5 (n-3) ‡	%	0.035	ND	ND	0.042	0.043	0.022	0.061	0.010

† The full names of identified fatty acids are listed in Table 1; ‡ Identified only in the control treatment without plant protection; ND—not detected.

**Table 3:** The main effects of agronomic treatments on the content of tocopherols in oat grain. Mean values marked with different letters are statistically significant according to Tukey’s test.

Chemical Properties	Unit	Plant Protection			Nitrogen Rate			Oat Morphotype	
		Control	Herbicide	Herbicide and Fungicide	N_0_	N_60_	N_120_	Hulled Oats	Hulless Oats
Tocopherols									
α-T	mg 100 g^−1^	1.492 a	1.003 ab	0.951 b	1.314	0.959	1.174	0.924 b	1.374 a
β-T	mg 100 g^−1^	0.203	0.169	0.287	0.231	0.229	0.198	0.191 b	0.248 a
γ-T	mg 100 g^−1^	1.529	1.204	1.327	1.678 a	1.30 ab	1.083 b	1.083 b	1.624 a
δ-T	mg 100 g^−1^	0.225	0.138	0.186	0.277	0.136	0.136	0.194	0.172
Total T	mg 100 g^−1^	3.448 a	2.507 b	2.751 b	3.500 a	2.621 b	2.584 b	2.388 b	3.416 a

**Table 4:** The main effects of agronomic treatments on the content of essential mineral elements in oat grain. Mean values marked with different letters are statistically significant according to Tukey’s test.

Chemical Properties	Unit	Plant Protection			Nitrogen Rate			Oat Morphotype	
		Control	Herbicide	Herbicide and Fungicide	N_0_	N_60_	N_120_	Hulled Oats	Hulless Oats
Major and minor essential mineral elements									
N	mg 100 g^−1^	1.888	1.627	1.608	1.535	1.587	2.002	1.697	1.719
P	mg 100 g^−1^	0.305 a	0.258 b	0.260 b	0.263	0.267	0.293	0.252 b	0.297 a
K	mg 100 g^−1^	0.43 ab	0.423 b	0.458 a	0.463 a	0.43 ab	0.418 b	0.438	0.437
Mg	mg 100 g^−1^	0.120	0.117	0.120	0.120	0.115	0.122	0.111 b	0.127 a
Ca	mg 100 g^−1^	0.162 a	0.125 ab	0.072 b	0.077 b	0.102 b	0.180 a	0.114	0.124
Trace essential mineral elements									
Cu	mg kg^−1^	2.383	2.100	2.450	2.733 a	1.950 b	2.25 ab	2.244	2.378
Fe	mg kg^−1^	52.1	50.8	52.6	48.9	50.3	56.3	57.9 a	45.8 b
Zn	mg kg^−1^	32.7 a	29.0 b	28.2 b	28.7	29.7	31.5	31.4 a	28.5 b
Mn	mg kg^−1^	26.1	25.6	26.1	23.9 b	27.3 a	26.7 ab	27.2 a	24.7 b

## Data Availability

The data presented in this study are available on request from the corresponding author. The data are not publicly available due to privacy restrictions.
